# Waterlogged Conditions Influence the Nitrogen, Phosphorus, Potassium, and Sugar Distribution in Sago Palm (*Metroxylon sagu* Rottb.) at Seedling Stages

**DOI:** 10.3390/plants11050710

**Published:** 2022-03-07

**Authors:** Aidil Azhar, Koki Asano, Daisuke Sugiura, Mana Kano-Nakata, Hiroshi Ehara

**Affiliations:** 1 Graduate School of Bioagricultural Sciences, Nagoya University, Nagoya 464-8601, Japan; asano.koki@j.mbox.nagoya-u.ac.jp (K.A.); daisuke.sugiura@gmail.com (D.S.); 2 School of Vocational Studies, IPB University, Bogor 16128, Indonesia; 3 International Center for Research and Education in Agriculture, Nagoya University, Nagoya 464-8601, Japan; mnakata@agr.nagoya-u.ac.jp; 4 Applied Social System Institute of Asia, Nagoya University, Nagoya 464-8601, Japan

**Keywords:** carbohydrate concentration, *Metroxylon sagu* Rottb., N, P, K concentration

## Abstract

Sago palm (*Metroxylon sagu* Rottb.) grows in well-drained mineral soil and in peatland with high groundwater levels until complete submersion. However, the published information on nutrient uptake and carbohydrate content in sago palms growing under waterlogging remains unreported. This experiment observed sago palm growth performance under normal soil conditions (non-submerged conditions) as a control plot and extended waterlogged conditions. Several parameters were analyzed: Plant morphological growth traits, nitrogen, phosphorus, potassium, and sugar concentration in the plant organ, including sucrose, glucose, starch, and non-structural carbohydrate. The analysis found that sago palm morphological growth traits were not significantly affected by extended waterlogging. However, waterlogging reduced carbohydrate levels in the upper part of the sago palm, especially the petiole, and increased sugar levels, especially glucose, in roots. Waterlogging also reduced N concentration in roots and leaflets and P in petioles. The K level was independent of waterlogging as the sago palm maintained a sufficient level in all of the plant organs. Long duration waterlogging may reduce the plant’s economic value as the starch level in the trunk decreases, although sago palm can grow while waterlogged.

## 1. Introduction

Variability in the period and duration of precipitation causes major issues for agricultural practice in some areas, especially where waterlogging occurs. Abiotic stress caused by waterlogging is a major impediment to crop growth. Due to waterlogging, the low O_2_ level in soil restricts water and nutrient uptake by roots. O_2_ is the acceptor of the electron in mitochondrial electron transport. Therefore, low O_2_ causes respiration to shift metabolism from the aerobic to anaerobic mode [1,2]. When the water and nutrient distribution from roots to leaflets is inhibited, the leaf photosynthetic performance will be automatically restricted.

In Indonesia, sago palm (*Metroxylon sagu* Rottb.) naturally grows in tidal swamps and lowlands. It is generally cultivated in peatland with high soil water levels and is susceptible to face waterlogging in long, severe rainy seasons. However, unlike other crops, sago palm can adapt to high groundwater levels [3], even submergence. In our previous study, we found that within a certain period of waterlogging, sago palm maintained the higher net photosynthetic rate than in normal soil water conditions. However, the net photosynthetic rate decreased by 23% after 2 months of continuous waterlogging [4].

Sago palm can grow in areas without a proper water drainage system or any soil improvement [5] as the root can interact symbiotically with commercial arbuscular mycorrhizal fungi (AMF) [6]. While studies have reported that waterlogging inhibited macronutrient uptake of oil palm [7] and reduced sugar concentration in sugarcane [8], the distribution pattern of nutrient concentration and carbohydrate distribution of sago palm under extended continuous waterlogging remains unreported.

Generally, sago palm grows wild or with minimal cultivation practices and fertilizer application. A previous study reported that applying N fertilizers two times within 1 year had no impact on plant height, leaf formation or N foliar concentration of sago palm [9]. This experiment was designed to evaluate the effect of waterlogging on nitrogen, phosphorus, potassium, and sugar concentration in sago palm. We hypothesized that prolonged and permanent waterlogging would reduce nitrogen, phosphorus, potassium (N, P, and K), and sugar concentration in the sago palm organs. 

## 2. Results

### 2.1. Morphological Growth Traits and Root Morphology for Adaptation

The plants grown under 107 days of waterlogging showed a lower growth trend in plant height, area of newly developed leaves, leaf dry matter weight, and petiole dry weight, although there was no significant effect of waterlogging upon shoot growth. Conversely, the sago palm tends to have higher root dry matter weight with waterlogging in all of the three (main, secondary, and fine) root types (Table 1).

**Figure 1:**
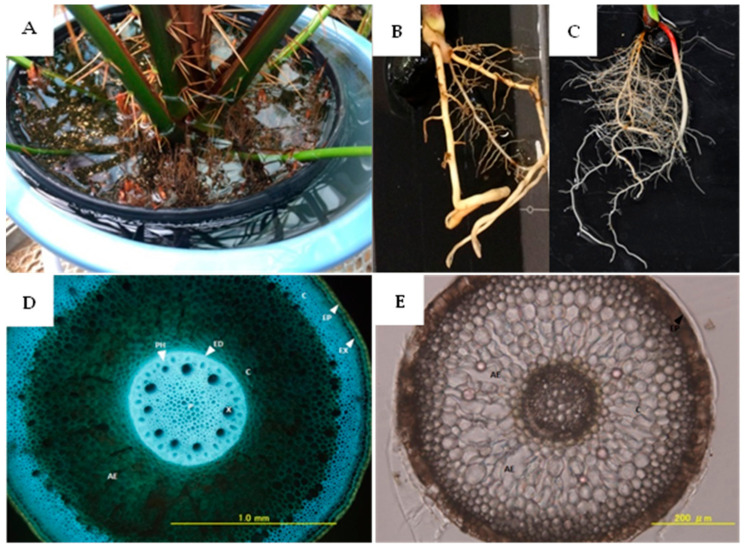
(**A**) Aerial root (pneumatophore) of sago palm seedlings. (**B**) Roots of sago palm seedlings under normal soil water condition and (**C**) waterlogging. (**D**) Aerenchyma tissues in thick (>2 mm) and (**E**) thin pneumatophore (<2 mm).

Under waterlogged conditions, sago palm produced many pneumatophores (aerial roots) that emerged from the soil surface (Figure 1A). Aerenchyma also developed in thick and thin pneumatophores (Figure 1D,E). Moreover, the root growth was more vigorous under waterlogging than under normal conditions. With waterlogging, the roots were visually confirmed to have higher number of fine roots (Figure 1B,C).

### 2.2. Sugar Concentration in Petioles and Roots

**Figure 2:**
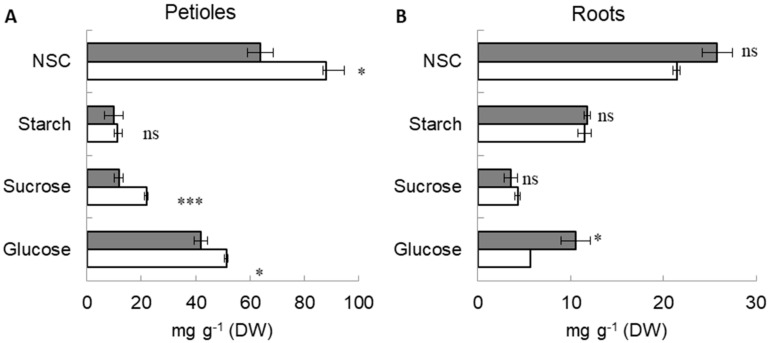
Glucose, sucrose, starch, and NSC concentration in sago petioles (**A**) and roots (**B**) under control (empty bars) and waterlogging (filled bars); ^ns^ indicates no significant difference. * and *** indicate significant levels at 0.05 and 0.001 probability levels, respectively, according to the Student *t*-test. Data correspond to the mean value and standard error (*n* = 3).

Under waterlogged conditions, the sago palm petioles were found to have lower sugar concentration in terms of glucose, sucrose, and non-structural carbohydrate (NSC) (Figure 2A). However, under the same conditions, the roots had higher concentration of glucose and NSC (Figure 2B).

**Figure 3:**
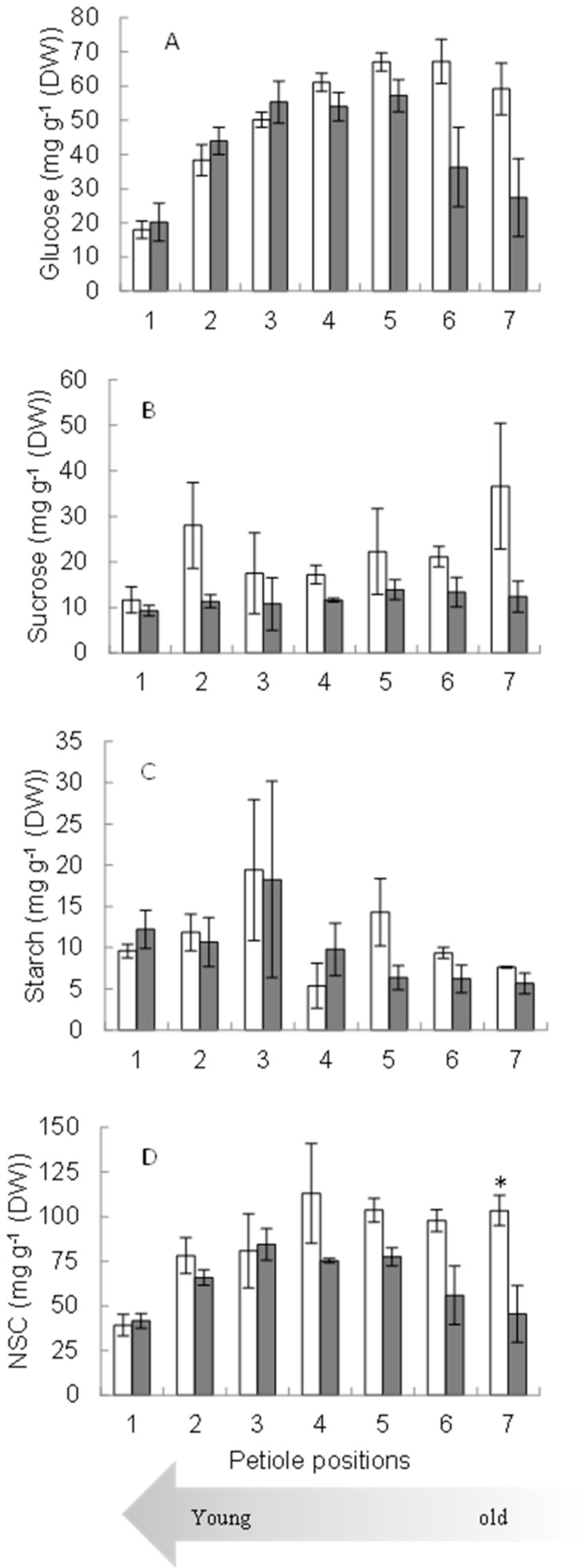
Glucose (**A**), sucrose (**B**), starch (**C**), and NSC (**D**) concentration at different petiole positions under control (empty bars) and waterlogged (filled bars) conditions. The numbers in *X*-axis mark the position of the leaf from the terminal down; * indicates the significant level at 0.05 probability level according to the Student *t*-test. Data correspond to the mean value and standard error (*n* = 3).

Under waterlogged conditions, the plants showed a more significant trend in glucose concentration in younger petioles (petiole numbers 1, 2, and 3). However, the trend of glucose concentration sharply decreased (Figure 3A) with the increasing age of the petioles (petiole numbers 4, 5, 6, and 7). A similar trend appeared in NSC concentration: Waterlogging reduced NSC concentration sharply in older petioles (Figure 3D). The trend of sucrose and starch concentration was also lower under the waterlogged condition than under the normal (control) condition (Figure 3B,C).

### 2.3. Nutrient Concentration in Each Plant Organ

**Figure 4:**
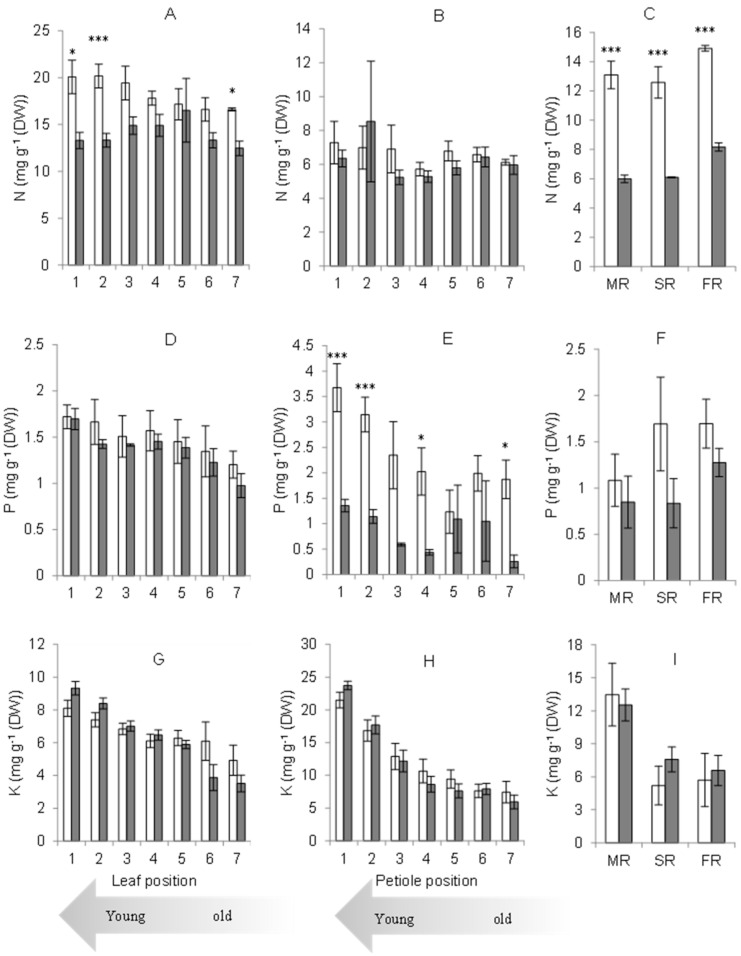
N, P, and K concentration in leaflets (**A**,**D**,**G**), petioles (**B**,**E**,**H**), and roots (MR: Main root, SR: Secondary root, and FR: Fine root) (**C**,**F**,**I**) under control (empty bars) and waterlogging (filled bars) conditions. The numbers in *X*-axis mark the position of the leaf from the terminal down; * and *** indicate the significant levels at the 0.05 and 0.001 probability levels, respectively, according to the Student *t*-test. Data correspond to the mean value and standard error (*n* = 3).

The leaflet provided stronger sinks for N than the petiole or root. N concentration in leaflets varied with the position of the leaves, which reflected the leaves’ age. N was predominantly distributed to the younger mature leaflets in the control plot. However, with waterlogging, N concentration in younger leaflets was low (Figure 4A). N concentration among the petiole positions was not affected by waterlogging (Figure 4B). Waterlogging reduced N concentration in roots. The trend in the N concentration pattern was higher in fine roots (FR) than in secondary (SR) and main roots (MR) under both normal and waterlogged conditions (Figure 4C).

Whereas the leaf was the major sink for N, the petiole was the major sink for P distribution under normal conditions. P level in the leaflet was not affected by waterlogging (Figure 4D). However, P concentration in the petiole decreased under waterlogging. The reduction of P concentration due to waterlogging was found in all of the petiole positions, from the oldest to the youngest petiole (Figure 4E). P distribution in roots was not significantly affected under waterlogged conditions, although a lower trend of P concentration was found with waterlogging (Figure 4F). 

K concentration was not affected by waterlogging in leaflets, petioles or roots. K was distributed more to younger leaflets and petioles, and the petiole was the most significant sink for K, followed by roots. With waterlogging, the K distribution to younger leaflets and petioles was higher than under normal conditions. In roots, the higher K concentration was found in the main root (Figure 4G–I).

## 3. Discussion

In waterlogged conditions, sago palm produced numerous pneumatophores with many aerenchyma tissues inside. The main pathways for oxygen supply to roots are through intercellular gas spaces and aerenchyma in the shoot–root continuum. In waterlogged conditions or oxygen-deficient soils, the above-ground part of the plant, which has direct contact with the atmosphere, becomes the primary source of oxygen supply to the below-ground portion of plant organs [10]. It has been reported that sago palm avoids excessive water stress by developing a lysigenous aerenchyma in the root, which transports oxygen from the above-ground part to the underground part [11]. A lysigenous aerenchyma is an intercellular space formed by the loss of adjoining tissue cells as a means of aeration during growth under waterlogged conditions [12]. Thereby, plants still have energy that allows them to absorb nutrients.

Previous research found that high groundwater levels increase sago palm’s photosynthetic rate [4], as well as shoot and root dry matter weight [13]. However, in this research, we found that the trend of shoot dry matter weight in plants was lower under extended waterlogging, but the trend of root matter weight was higher. 

With continuous waterlogging, the sucrose, glucose, and non-structural carbohydrate concentration in petioles decreased. As the primary product of photosynthesis, sucrose is used as a transport and storage molecule. When the photosynthetic rate was reduced due to waterlogging [13], the sucrose level decreased as the plants satisfied their needs using the stored sugar [14]. Sago palm accumulated higher glucose and NSC concentration in the roots under waterlogging than under normal conditions. This mechanism can be considered one of the adaptive mechanisms of sago palm, which responds to an oxygen-deficient environment under prolonged waterlogging [15]. Similar information has been reported for a soybean hypoxia tolerant genotype in which, due to hypoxia, sugar concentration increased in roots and decreased in nodules [16]. Accumulation of soluble sugars in the roots of plants under soil flooding has been reported as a survival strategy in response to this stress [17,18].

Waterlogging reduced N concentration in roots. It can be seen from the significant reduction of N concentration in all of the root types of sago palm grown under waterlogged conditions. The reduction of N level in roots might be caused by the inhibition of ATP synthesis under low oxygen conditions, which results from the change in plasma membrane H^+^ ATPase [19]. Generally, nutrient uptake is energy-dependent, while reducing ATP availability for pumps reduces their uptake [20]. With a low soil oxygen level, root oxygen supply becomes dependent on O_2_ supply from shoots. Therefore, nutrient uptake from soil may continue [21]. The reduction of N level also occurred in younger sago leaflets. The transfer of N from older to younger leaflets was slower. The reduction of leaf N concentration may lead to a reduction in leaf chlorophyll concentration [22], maximum electron transport rate, and enhanced efficiency of the light-harvesting complex in photosystem II [23]. Consequently, the net assimilation rate was also affected [24]. A study of young Populus × canescens tree response to hypoxia reported that N concentration was abundant in fine roots, and the allocation of N dropped by 50% due to hypoxia conditions [25]. The decline of O_2_ concentration due to waterlogging negatively affects mitochondrial respiration, and a lack of energy in the cells occurs. The energy consumption in ATP for N and other nutrient uptakes will be severely inhibited [26]. 

With waterlogging, P concentration was also affected in petioles, but not in leaves and roots. The reduction of P concentration may have resulted in a reduction in energy availability. As a central component of energy metabolism (ATP and Pi), P plays a vital role in macromolecular nucleic acids formation, ribosomes, phospholipids, and sugar phosphates [27]. The reduction of N uptake is less likely to be caused by the decrease in energy availability with P limitation.

This experiment found that the K level in the plant organ was not significantly affected by waterlogging. Sago palm seems to aggressively accumulate an adequate or higher K concentration under particular abiotic stress conditions. This is not confined to waterlogging. Previous studies reported that sago palm had adequate or even higher K concentration under salt stress conditions, with low pH and low nutrient supply in the culture solutions. The high NaCl level (342 mM) did not affect K concentration in the leaflet or petiole [28]. Moreover, another study reported that low pH levels in the culture solution (4.5 and 3.6) caused increased K concentration in sago palm petioles [29]. In our former unpublished work on sago palm response to different concentrations of Kimura-B culture solution, we found that sago palm accumulated higher K concentration in leaflets under low nutrient concentration (10%) than under higher nutrient concentration (50 and 100%) of Kimura-B culture solution. In our other unpublished study, we also found that K concentration in the sago palm grown in peat soil with low nutrient levels was comparatively higher than in mineral soil with higher soil nutrient levels.

## 4. Materials and Methods

### 4.1. Experimental Location and Materials

The experiment was conducted in an enclosed space with controlled air temperature (phytotron) at Nagoya University, Japan, from July to November 2017. During the study, no artificial light source was applied. Air temperature ranged from 29–33 °C, and relative humidity ranged from 50–60%, consistent with optimal air conditions for sago palm photosynthetic performance [24]. Two-year-old sago palm seedlings of the *Tuni* folk variety (with spiny petioles) grown in 5 L plastic pots were subjected to two different growing conditions: Control (water was capable of draining through the holes under the pots, and the plants were watered every day until the soil reached water capacity) and waterlogging (water level was maintained above the soil surface) (*n* = 3). The plant materials were obtained in seed form from Sentani District, Jayapura, Indonesia. After 107 days in the waterlogged condition, sago palm seedlings were harvested for nutrients and sugar analysis. The sago seeds obtained from Sentani District, Jayapura, Indonesia, were germinated individually in pots (diameter 115 mm and height 184 mm) filled with vermiculite. After 1.5 years, the seedlings were transplanted to bigger pots (5 L) filled with commercial black soil (Protoleaf, Tokyo, Japan). They were subjected to inundation treatment when they had reached at least 2 years of age and had 12 fully-developed leaves.

### 4.2. Morphological Growth Traits

Morphological growth traits of sago palm, including (1) plant height, (2) newly developed leaflet area, (3) dry leaflet weight, (4) dry petiole weight, and (5) dry root weight, were measured at the end of the experiment. The height of the plant was measured from the lower part of the plant until the leaf tip. The newly developed leaflet area was measured using the portable leaf area measuring device LI-3000C (Lincoln, NE, USA). The root, petiole, and leaflet were dried at 70 °C until the constant dry weight could be measured.

Pneumatophore and aerenchyma observations were visually carried out on the 2-year-old sago seedlings, without counting the number of pneumatophore aerenchyma tissue. The root cross-sections of thick basal pneumatophore (>2 mm) and thin pneumatophore (<2 mm) were obtained using the Vibratomes Leica VT 1200S (IL, United States). The root-crossed sections were viewed and photographed with an UV microscope.

### 4.3. Carbohydrate Analysis

Carbohydrate concentration was analyzed in petioles and roots. Five milligrams of ground samples were used for sugar analysis. In addition, 80% of ethanol was used to extract glucose, sucrose, and starch at 78.5 °C for 10 min two times. Starch was determined by the precipitation, and the supernatant was concentrated centrifugally for 3 h (CC-105; Tomy Seiko, Tokyo, Japan). Non-structural carbohydrate (NSC) concentration was obtained from combined values of glucose, sucrose, and starch concentration. The detailed description of sugar analysis was explained in our previous study [13]. 

### 4.4. Nitrogen, Phosphorus, and Potassium Analysis

The concentration of nitrogen, phosphorus, and potassium was analyzed on the sago seedlings’ leaflets, petioles, and roots. The root was classified into three different areas: The main root (roots that grow from the surface of the bark, then extend to the growing medium, diameter >2 mm), secondary root (roots that grow from main roots, diameter 2 mm), and fine root (roots that grow from secondary roots, diameter <2 mm). All of the samples were dried in the oven for 3 days at 70 °C to achieve the constant dry matter weight. The samples were ground for nutrient and sugar analysis. One milligram of each sample was used to analyze the N concentration utilizing a CN analyzer (Vario EL, Elementar Analyzensysteme GmbH, Hanau, Germany). The concentration of P and K was analyzed by digesting 0.5 g of ground samples using the Kjeldahl digestion method with H_2_SO_4_ and H_2_O_2_.

The concentration of P was analyzed by adding 4% of ammonium molybdate, potassium antimonyl tartrate, 5 N sulfuric acid, and *L*-ascorbic acid to 0.25 mL of digested sample solution. The phosphorus concentration was analyzed using a spectrophotometer (UV-1800 Shimadzu, Kyoto, Japan) at 710 nm of wavelength. A flame photometer (BWM Technologies Ltd. Newbury, UK) was used to estimate K concentration in the digested solution [13].

### 4.5. Statistical Analysis 

Data from each parameter are presented as mean ± SE. A Student *t*-test was performed using MS excel (Microsoft Windows Version 6.3) to test for differences in all of the parameters. 

## 5. Conclusions

The findings of this study suggested that although under continuous waterlogging the growth trend of the above-ground part of the plant was low, waterlogging induced the higher trend of root morphological growth for adaptation to the waterlogged condition. Waterlogging increased the pattern of sugar concentration in roots as the glucose and NSC concentration in the roots increased. Continuous waterlogging also reduced the N and P concentration in sago palm seedlings. It reduced nitrogen and phosphorus concentration in leaflets and petioles, respectively. However, the K concentration was not affected by continuous waterlogging. Further studies on mature sago palm in waterlogged areas are needed to evaluate sago trunks’ growth speed and carbohydrate concentration. 

## Figures and Tables

**Table 1:** Growth traits of sago palm seedlings for each water treatment (control and waterlogging).

Growth Traits	Control	Waterlogging
Area of new developed leaflet (m^2^)	0.35 ± 0.073	0.34 ± 0.032
Leaf dry weight (g)	9.71 ± 1.07	8.15 ± 0.59
Petiole dry weight (g)	20.95 ± 1.58	18.79 ± 0.91
Plant height (cm)	122 ± 6.64	117.63 ± 1.85
Main root dry weight (g)	17.35 ± 2.23	17.96 ± 2.23
Secondary root dry weight (g)	7.78 ± 1.20	11.07 ± 1.78
Fine root dry weight (g)	13.93 ± 3.09	18.99 ± 1.63
Total root dry weight (g)	39.06 ± 5.62	48.03 ± 5.04

## Data Availability

Not applicable.
